# Stabilisation and Growth of Metastable Form II of Fluconazole in Amorphous Solid Dispersions

**DOI:** 10.3390/pharmaceutics12010012

**Published:** 2019-12-20

**Authors:** Maciej Nowak, Maciej Gajda, Przemysław Baranowski, Patrycja Szymczyk, Bożena Karolewicz, Karol P. Nartowski

**Affiliations:** 1 Department of Drug Forms Technology, Wroclaw Medical University, Borowska 211A, 50-556 Wroclaw, Poland; maciej.nowak@umed.wroc.pl (M.N.); maciej.gajda@umed.wroc.pl (M.G.); przemyslaw.baranowski@umed.wroc.pl (P.B.); bozena.karolewicz@umed.wroc.pl (B.K.); 2 Centre for Advanced Manufacturing Technologies (CAMT/FPC), Wroclaw University of Science and Technology, Lukasiewicza 5, 50-371 Wroclaw, Poland; patrycja.e.szymczyk@pwr.edu.pl

**Keywords:** polymorphism, fluconazole, Soluplus, ASD, stability, spray drying, amorphous solid dispersion

## Abstract

The crystallisation of metastable drug polymorphs in polymer matrices has been reported as a successful approach to enhance the solubility of poorly water-soluble drug molecules. This can be achieved using different polymers, drug to polymer ratios and formulation techniques enabling the formation of stable nuclei and subsequent growth of new or metastable drug polymorphs. In this work we elucidated the polymorphism behaviour of a model compound fluconazole (FLU) embedded in solid dispersions with amorphous Soluplus^®^ (SOL) obtained using spray drying and fusion methods. The effect of humidity on the stability of FLU in the obtained dispersions was also evaluated. FLU at a drug content below 40 wt. % stayed amorphous in the dispersions prepared using the fusion method and crystallised exclusively into metastable form II at a drug content above 40 wt. % and 70% relative humidity (RH) conditions. In contrast, a mixture of forms I, II and hydrate of FLU was detected in the spray dried formulations after 14 days of storage at 40 °C/40% RH, with preferential growth of thermodynamically stable form I of FLU. This study highlights the importance of preparation techniques and the drug:polymer ratio in the formulation of amorphous solid dispersions and provides further understanding of the complex crystallisation behaviour of amorphous pharmaceuticals encapsulated in the polymer matrixes.

## 1. Introduction

To date, several methods have been proposed to enhance the solubility and dissolution rate of poorly water-soluble drug candidates, which are approximated to be 40% of the new chemical entities (NCE) in the industrial development. Examples include micronisation [1], complexation [2], polymorphs [3] and cocrystals [4] formation, chemical modification (e.g., prodrugs) [5] of APIs (Active Pharmaceutical Ingredients) or the formulation of drug solid dispersions in hydrophilic polymer matrices [6]. The drug molecules can be incorporated in the polymer matrix in either amorphous or crystalline form, which determines both the dissolution performance and physicochemical stability of the obtained formulations. Amorphous solid dispersions (ASD) composed of an amorphous API dispersed in an amorphous polymer matrix are well-established in pharmaceutical drug delivery due to the (i) increased drug dissolution rate, (ii) improved wettability of the powder, (iii) reduction of the particle size and (iv) stabilization of an amorphous form of a drug in the polymer matrix [7]. The major limitation of the wide use of the ASDs in pharmaceutical drug delivery is their thermodynamic instability, resulting in API crystallisation during storage, which may affect the dissolution rate of the drug compromising its therapeutic benefit. The long term stability of the drug:polymer ASDs depends on the structure and properties of the drug and polymer, the content of the API in the formulation and the preparation technique. Solid dispersions composed of a crystalline API incorporated in the amorphous (or semi crystalline) polymer matrices are important alternatives to amorphous systems as they can display an increased dissolution rate as compared to formulations based on a neat crystalline drug and prolonged physicochemical stability as compared to ASDs. Crystallisation and stabilisation of metastable drug polymorphs in polymeric matrices are of increasing importance in pharmaceutical drug delivery as promising methods to increase solubility of poorly water-soluble molecules [6,8] and as a tool in intellectual property management. Recently, Censi and Di Martino reviewed the practical aspects of the drug polymorphism effect on bioavailability and stability of poorly water soluble molecules including several market examples of APIs formulated as metastable polymorphs [9]. Furthermore, the use of polymer matrices in crystallisation processes is a vital area of research in crystal engineering and materials science as it enables access to different nucleation and crystallisation pathways, which may result in the formation of new polymorphs. For example, the metastable form of probucol (form II) selectively crystallised in polyacrylic acid (PAA) or polyethylene oxide (PEO) was shown to have a significantly higher drug release as compared to amorphous solid dispersions of probucol in polyvinyl pyrrolidone (PVP) [10]. Docoslis et al. observed preferential crystallisation and prolonged stabilisation of better soluble, metastable heterochiral form of nimodipine (modification I) formulated as solid dispersion with PEG 4000 [11]. Zhu et al. reported selective crystallisation of the metastable form of chlorpropamide (form B) in PEG 3350 at 20 wt. % drug content in the solid dispersion [12]. Similarly, Martınez-Oharriz observed selective crystallisation of the metastable form of diflunisal (form III) in PEG 4000 solid dispersions, which was related to the drug content in the formulation and preparation method i.e., fusion or solvent coprecipitation [13]. Recently, Telford et al. reported on melt crystallisation and stabilisation of highly unstable paracetamol form III using β-1,4-saccharides (lactose monohydrate and HPMC) as excipients [14]. Furthermore, indomethacin and topiramate incorporated in the PEG matrices were shown to crystallise into new metastable polymorphs in the formulations [15,16]. Several examples of dissolution rate enhancement and excipient induced formation of metastable forms I and II of carbamazepine in PEG, PVP and phospholipids have also been described [17,18,19]. In our recent studies we showed selective crystallisation and stabilisation of metastable tolbutamide form V and indomethacin form V encapsulated in mesoporous silicas MCM-41 and MCF [20,21].

In this work we investigated the crystallisation behaviour of the model drug fluconazole (FLU) incorporated within amphiphilic polymer matrix based on polyvinyl caprolactam–polyvinyl acetate–polyethylene glycol graft copolymer (SOL, Soluplus^®^) at a drug content from 10 to 60 wt. % in the formulations. The solid dispersions were prepared using a fusion and spray drying method, both being used industrially for the preparation of API:polymer solid dispersions [22,23]. The phase of the drug within the formulation directly after preparation and after storage at 40 °C and 40/70% RH was assessed using the combined application of powder X-ray diffraction (PXRD), Fourier transformed infrared spectroscopy and thermal methods (differential scanning calorimetry, DSC and thermogravimetric analysis, TGA). The effect of the structural changes of the formulations upon storage of the dissolution profile of the drug was also discussed for the spray dried formulations.

With its well described polymorphic landscape, highly flexible structure and the presence of seven hydrogen bond acceptor groups and one hydrogen bond donor, fluconazole is a very good model for investigating the crystallisation processes in polymer matrices. Nine polymorphs, several cocrystals and solvates (water, ethyl acetate, benzene, acetone) of fluconazole have been reported to date [24,25,26,27] and the formulation of FLU solid dispersions with PLGA, HPMC, PVP and Chitosan were shown to increase solubility of the drug [28,29]. Moreover the DSC and PXRD data presented by Papageorgiou et al. for the FLU dispersions in HPMC, PVP and Chitosan indicated the formation of amorphous FLU at 20 wt. % drug content in the formulations but crystallisation of FLU hydrate at higher drug loadings [29], which significantly differed from our observations.

Therefore, the aim of this work was to understand the effect of the polymer matrix, preparation method and drug content on the crystallisation processes of model FLU embedded in SOL polymer with the aid of complementary analytical techniques sensitive to structural changes and local interactions of molecules in the formulations. This is of contemporary importance from both academic and industrial research in the fields of crystal engineering, materials and preformulation science.

## 2. Materials and Methods

### 2.1. Materials

Pharmaceutical grade fluconazole (FLU) and Soluplus^®^ (SOL) were kindly donated by P.P.F. “Hasco-Lek” (Wrocław, Poland) and BASF (Ludwigshafen, Germany). Acetonitrile, dichloromethane and methanol HPLC grade were purchased from J. T. Baker (Deventer, Netherlands). The other chemicals and reagents used in this study were of analytical grade.

### 2.2. Formation of Fluconazole (FLU) Hydrate and FLU Form II

FLU hydrate (2 g) was formed by suspending FLU form I in distilled water under stirring for 24 h at room temperature. The obtained crystals were filtered and dried over night at room temperature. FLU form II (2 g) was obtained by crystallisation from an amorphous FLU at 100 °C for 2 hr. In order to unify the particle sizes of all investigated FLU polymorphs (FLU form I—commercial, FLU form II and FLU hydrate) the materials were grained using a mortar and pestle and sieved through a 80 µm sieve prior to the dissolution study. The phase of the resulting materials was assessed using PXRD and FTIR prior to dissolution studies.

### 2.3. Preparation of Solid Dispersions by the Fusion Method

During preparation of FLU:SOL solid dispersions drug and polymer in the 10:90; 20:80; 30:70; 40:60; 50:50 or 60:40 FLU to SOL ratio were thoroughly mixed using mortar and pestle and the resultant powder was transferred to aluminium pans and heated to 145 °C in the oven i.e., above the melting point of FLU. Samples were kept at this temperature for 10 min to ensure complete melting and left at room temperature (21.0 ± 2 °C) to solidify. The thermal stability of both materials was investigated using TGA before the experiment. The obtained formulations were stored in a vacuum desiccator at 30% RH in tightly closed amber glass Duran^®^ bottles (Mainz, Germany) prior to further analysis.

### 2.4. Preparation of Solid Dispersions by the Spray Drying Technique

Six FLU:SOL formulations were prepared using the spray drying method with FLU content in the final formulation of 10, 20, 30, 40, 50 and 60% *w*/*w*. To prepare 10 g of each batch accurately, weighed amounts of FLU and SOL were dissolved in a 50/50 *v*/*v* mixture of dichloromethane/methanol to obtain 5% *w*/*v* solutions. The solution was spray dried in a closed loop using a Mini Spray Dryer B-290 coupled with a Dehumidifier B-296 and an Inert-loop B-295 (Büchi, Flawil, Switzerland). The feeding solutions were atomized through a two-fluid nozzle, whose inner diameter was 0.7 mm. Ultra-high purity nitrogen was used as spray gas at a pressure of 5 bar. The spray drying process was carried out under the following conditions: peristaltic pump rate 5.0 mL·min^−1^, N_2_ flow rate 600 L·h^−1^, aspiration rate 32 m^3^·h^−1^, inlet temperature 80 ± 1 °C and outlet temperature 50 ± 1 °C. The spray dried particles were separated in a cyclone, collected and stored until further analysis. The obtained formulations were stored in a vacuum desiccator at 30% RH in tightly closed amber glass Duran^®^ bottles (Mainz, Germany) prior to further analysis.

### 2.5. Powder X-ray Diffraction (PXRD)

The PXRD analysis was conducted using a D2 PHASER diffractometer (Bruker AXS, Karlsruhe, Germany) with a LynxEye detector using Cu Kα radiation (1.5418 Å). The data were collected with Bragg–Brentano (*θ*/2*θ*) horizontal geometry between 5° to 50° 2*θ*. A step size of 0.016° 2*θ* was used with 0.5 sec/step. The optics of the D2 PHASER diffractometer was a Soller slit module system with 2.5°, a divergence slit with 0.6 mm, an air-scatter screen with 1 mm and a Ni filter. The X-ray tube operated at 30 kV and 10 mA.

### 2.6. Fourier-Transform Infrared Spectroscopy (FTIR)

The FTIR spectra were recorded using a Nicolet iS50 spectrometer (Thermo Scientific, Waltham, MA, USA) equipped with an attenuated total reflectance (ATR). The spectra were recorded over a wavelength of 400 cm^−1^ to 4000 cm^−1^ at 32 scans per sample and a resolution of 4 cm^−1^.

### 2.7. Differential Scanning Calorimetry (DSC)

Differential scanning calorimetry analysis was carried out using a DSC 214 Polyma instrument (Netzsch, Selb, Germany) equipped with an IntraCooler. The samples (5 ± 0.5 mg) were weighed to aluminium pans (25 µL) and closed with pierced lids. Each sample was heated to 150 °C at a heating rate of 5 °C·min^−1^ and held at this temperature for 10 min before cooling to −50 °C at the 5 °C·min^−1^ cooling rate. Subsequently, the sample was reheated to 160 °C at a heating rate of 5 °C·min^−1^. The analysis was performed in a nitrogen atmosphere (50 mL·min^−1^). An empty pan closed with a pierced lid was used as a reference. The DSC peak area and transition temperatures were determined using the Netzsch Proteus Analysis software 7.1.0 (16.10.2017). The DSC instrument was calibrated using six melting standard samples from a calibration set 6.239.2-91.3 supplied by Netzsch.

### 2.8. Thermogravimetric Analysis (TGA)

The TGA curves were recorded using a Netzsch TG 209 F1 Libra Thermobalance (Netzsch, Selb, Germany). The mass of the analysed materials was 10.0 ± 0.5 mg. The materials were placed in aluminium oxide crucibles (Al_2_O_3_) and heated from 25 to 800 °C with a heating rate of 5 °C·min^−1^ in nitrogen atmosphere (50 mL·min^−1^). The obtained curves were analysed using the Netzsch Proteus Analysis software.

### 2.9. Dissolution Study

The dissolution studies were performed using USP type II dissolution test apparatus SR8-PLUS (Hanson, Chatsworth, CA, USA) with a mini paddle and small volume vessel (Hanson, Chatsworth, CA, USA) in non-sink conditions. The samples, equivalent to 50 mg FLU, were packed in gelatine capsules prior to analysis. Each capsule was placed inside a sinker (Japanese Pharmacopeia Basket Sinker) to avoid flotation and then in a dissolution vessel containing 150 mL high-purity water maintained at 37 ± 0.5 °C and stirred at 100 rpm. The 3 mL samples were withdrawn through the in line 0.45 μm filters (Quality Lab Accessories LLC, Telford, PA, USA) at 7.5; 15; 22.5; 30; 45; 60; 90; 120; 180; 240; 300 and 360 min and a volume of withdrawn medium was refilled. After filtration through 0.2 μm filter (Macherey-Nagel, Düren, Germany), the concentration of FLU was determined using HPLC.

### 2.10. High Pressure Liquid Chromatography (HPLC)

An Infinity 1260 system (Agilent Technologies, Waldbronn, Germany) fitted with a quaternary pump, a high performance auto sampler with a thermostat, a thermostatted column compartment and a diode-array detector were used to determine the content of dissolved FLU in dissolution studies via HPLC analysis based on the method described in the USP 32. Separation was conducted on the Zorbax Stable-Bond C18 column (150 × 4.6 mm, 5 µm). A reverse-phase HPLC assay was carried out using an isocratic system with a flow rate of 0.7 mL min^−1^, a column temperature of 40 °C and a mobile phase of water:acetonitrile 80:20 *v*/*v*. Analyte was identified by a UV-Vis detector at 260 nm. External standards of FLU were used to obtain calibration curves in the mobile phase. The linear correlation coefficient (*r*^2^) was greater than 0.99 in the range of 0.55–408.0 μg·mL^−1^.

### 2.11. Scanning Electron Microscopy (SEM)

The materials were stuck onto carbon rings and covered with gold and palladium (60:40; sputter current—40 mA; sputter time—50 s) using a Quorum sputter coater (Quorum Technologies Ltd., Laughton, UK) and examined under a Zeiss EVO MA25 scanning electron microscope (Carl Zeiss, Jena, Germany).

### 2.12. Stability Studies

The accelerated stability studies were performed in a climate chamber KBF-LQC 240 (Binder, Tuttlingen, Germany). Raw materials and solid dispersions prepared by the fusion method were kept in open glass vials at 40 °C and 40 and 70% relative humidity (RH) for 14 days. Solid dispersion prepared by spray drying were kept at 40 °C and 40% RH for 14 days. The changes in the materials crystallinity and the in vitro drug release were investigated after 7 and 14 days.

### 2.13. True Density of FLU and SOL

The true density of amorphous FLU and SOL powders was determined using AccuPyc 1340 Gas pycnometer (Micromeritics, Norcross, GA, USA). Each sample was filled in a 10.0 cm^3^ sample cup and the weight of each sample was noted. A true density measurement was then carried out at an equilibration rate of 0.0050 psig·min^−1^ using a set of 10 purges. Calibration was performed prior to each run.

### 2.14. Theoretical Calculation of the Glass Transition Temperature (T_g_) of Fluconazole (FLU):SOL Binary Systems using the Gordon-Taylor Equation

The *T*_g_ of all synthesised FLU:SOL binary systems were theoretically predicted using the Gordon–Taylor equation as per the formula shown below (Equation (1)):

(1) $$T_{g\left(\mathrm{FLU}/\mathrm{SOL}\right)}=\frac{w_{\left(\mathrm{FLU}\right)}T_{g\left(\mathrm{FLU}\right)}+Kw_{\left(\mathrm{SOL}\right)}T_{g\left(\mathrm{SOL}\right)}}{w_{\left(\mathrm{FLU}\right)}+Kw_{\left(\mathrm{SOL}\right)}};\;K\approx \frac{T_{g\left(\mathrm{FLU}\right)}\rho_{\left(\mathrm{FLU}\right)}}{T_{g\left(\mathrm{SOL}\right)}\rho_{\left(\mathrm{SOL}\right)}}$$

where *T*_g(FLU)_ and *T*_g(SOL)_ are the glass transition temperatures of the neat components (*T*_g(FLU)_ = 30.2 °C and *T*_g(SOL)_ = 69.5 °C), *w*_(FLU)_ and *w*_(SOL)_ are the weight fractions of the drug and polymer in the system. *K* is calculated from the true density *(ρ)* and *T_g_* of neat FLU and SOL (*K* = 0.54). The constant *K* represents the ratio of the thermal expansion coefficient difference between a glassy state and a liquid state between the components FLU and SOL. The true densities of SOL (1.175 g·cm^−3^) and amorphous FLU (1.457 g·cm^−3^) were determined using a pycnometer (see Section 2.13 for details). Gordon and Taylor based their theory on two basic assumptions: volume additivity, i.e., an ideal volume of mixing and a linear change in volume with temperature [30]. The experimental values of *T*_g_ were determined for all synthesised materials using the second heating cycle in the DSC analysis performed according to the protocol described in Section 2.7. Differential scanning calorimetry (DSC).

## 3. Results and Discussion

### 3.1. Stability and Recrystalisation Behaviour of Amorphous Fluconazole

A commercially available form of FLU (form I) melted at 140.6 °C with a heat of fusion equal to 34.1 ± 0.5 kJ·mol^−1^ in agreement with previously published data. Frequently occurring hydrate of fluconazole undergoes thermal desolvation at 98.7 °C, which results in the formation of FLU form I. After melting FLU solidified as a glass (*T*_g_ = 30.2 °C) and crystalized to polymorphic form II of FLU on heating at 77.4 °C as confirmed using PXRD, FTIR and DSC measurements (ESI, Figures S1–S3). FLU form II subsequently melted at 135.3 °C with a heat of fusion determined as 34.6 ± 0.5 kJ·mol^−1^ (we used the nomenclature of FLU polymorphs proposed by Alkhamis et al. [24]). This was in agreement with the studies of Desai and Dharwadkar, who previously described thermal crystallisation of amorphous FLU [25]. The first crystals of the drug were formed at room temperature just one hour after the preparation, despite a relatively large difference between *T*_g_ of amorphous FLU (*T*_g_ = 30.2 °C) and its cold crystallisation temperature (*T*_cryst_ = 77.4 °C) determined using DSC (Figure S1), which could indicate long stability of the amorphous drug. Furthermore, the storage conditions of amorphous FLU determined the crystallisation outcome. Selective crystallisation of amorphous drug to FLU form II was observed at 40 °C/40% RH as confirmed using PXRD, FTIR and DSC analysis, while a mixture of FLU hydrate, form I and form II was detected at 40 °C/70% RH using PXRD and FTIR (ESI, Figures S1–S3).

### 3.2. Stabilisation and Crystallisation Behaviour of an Amorphous Fluconazole (FLU) in the SOL Polymer Matrix Prepared by Fusion Method

As formulation of API with polymers into amorphous solid dispersion may stabilize the amorphous state of the drug, we evaluated SOL as a promising polymer candidate for formulation of API:polymer amorphous solid dispersion with increased physicochemical stability.

The phase of FLU in solid dispersions prepared by the fusion method was evaluated immediately after preparation and after 14 days of accelerated stability studies. The PXRD patterns obtained directly after preparation of the materials displayed a broad ‘halo’ (Figure S4), characteristic of amorphous solids. The PXRD results were supported by FTIR studies, in which broadened vibrational bands of FLU were observed due to a lack of long-range ordering (Figure S5). The increase in FLU concentration within formulations resulted in increased intensity of the peaks attributed to an amorphous drug at 1500.6 cm^−1^, 1272.3 cm^−1^, 1137.3 cm^−1^, 965.7 cm^−1^, 849.5 cm^−1^, 677.4 cm^−1^, 652.0 cm^−1^, 616.2 cm^−1^, 525.2 cm^−1^, 514.2 cm^−1^ (vibrational band assignments based on [31], Table S1). Further understanding of the drug:polymer interactions within obtained solid dispersions was based on the analysis of the shifts of the IR peak positions observed in the spectra. The potential hydrogen bonds can exist between the FLU hydroxyl group (H donor) and SOL ester C=O and C–O groups, internal amid C=O group and C–O–C ether group (H acceptors) as well as between SOL hydroxyl group (H donor) and FLU triazole rings (H acceptors). With the increasing drug to polymer ratio, shifts of SOL peaks (2925.7 cm^−1^ to 2928.9 cm^−1^—C–H stretching, 1233.7 cm^−1^ to 1243.9 cm^−1^ and 1195.2 cm^−1^ to 1201.7 cm^−1^—ester C–O stretching) were observed suggesting possible interactions between FLU and SOL (see Figure S6 for comparison). Additionally, a change in the position of the IR peak of FLU difluorophenyl ring (C–C–C in plane bending, 675.4 cm^−1^ to 677.4 cm^−1^) was observed. The DSC curves of materials containing up to 50 wt. % of FLU did not show any events which could be assigned to crystallisation or melting (Figure S7). Broadened, low intensity endotherm starting above 10 °C was related to the glass transition and subsequent evaporation of residual, surface adsorbed water from the samples as confirmed using TG analysis (Figure S8). The adsorption of the atmospheric water during sample preparation and handling could be explained by high hygroscopicity of the polymer as recently reported by Lavra et al. for spray dried solid dispersions of efavirenz in SOL and Nowak et al. for tadalafil:SOL co-milled solid dispersions [32,33]. When the drug content in the formulation reached 60 wt. %, recrystallisation of amorphous FLU was observed on the DSC curve (Figure S7) as an exothermic event centred at 114.8 °C followed by the broad endotherm, which was related to the melting of FLU form II at 130.7 °C and subsequent recrystallisation and melting of FLU form I. This was supported by PXRD analysis (Figure S9) and agreed with the crystallisation behaviour of amorphous FLU (Figures S1–S3) and previously reported data by Desai and Dharwadkar [25]. The broadening and decrease in the melting point of FLU form II incorporated in the polymer matrix may be related to partial dissolution of a drug in the polymer matrix prior to melting, the formation of an eutectic mixture and/or the formation of nanosize crystals, all being frequently observed phenomena in crystalline drug:polymer solid dispersions [16,34,35].

DSC curves of the FLU:SOL solid dispersions obtained during the second heating cycle revealed single *T*_g_ per system, occurring between the glass transition temperatures of neat FLU (*T*_g_ = 30.2 °C) and SOL (*T*_g_ = 69.5 °C) (Figure S10). A single value of *T*_g_ recorded for all solid dispersions with 10 to 60 wt. % of FLU indicated miscibility of both components and existence of a single amorphous phase [36]. The experimentally determined *T*_g_ values of the materials with a drug content above 20 wt. % were lower as compared to theoretically calculated *T*_g_ values based on the Gordon-Taylor equation (negative deviation). This may indicate volume nonadditivity resulting from the nonideal mixing of FLU and SOL, which was most pronounced at drug contents above 40 wt. % (Δ*T*_g_ = −11 ± 2 °C; see ESI Figures S10 and S11) and may partially explain the crystallisation behaviour of FLU incorporated within SOL matrix at drug loadings above 40 wt. %.

**Figure 1:**
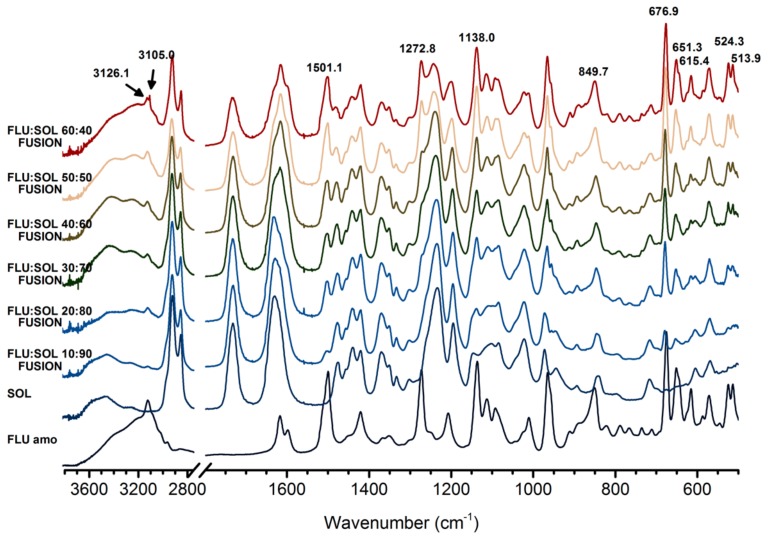
FTIR spectra of FLU:SOL solid dispersions prepared by the fusion method after 14 days of stability studies (40 °C, 40% RH).

FTIR spectra (Figure 1) of FLU:SOL formulations with drug content in the polymer matrix ranging from 10 to 50 wt. % obtained via the fusion method displayed broad peaks characteristic of amorphous solids which lack long range ordering after 14 days of storage at 40 °C/40% RH. The FTIR spectrum of the material with the highest drug content i.e., 60 wt. % of FLU displayed low intensity peaks of FLU form II (3126.7 cm^−1^, 3105.1 cm^−1^ assigned to triazole ring CH stretching), superimposed on the broad peaks of the amorphous FLU and SOL. Similarly, PXRD patterns of the materials after 14 days of accelerated stability studies (40 °C/40% RH) displayed a broad halo (Figure S12) characteristic of amorphous materials. This indicated increased stability of amorphous FLU incorporated within a polymer matrix.

**Figure 2:**
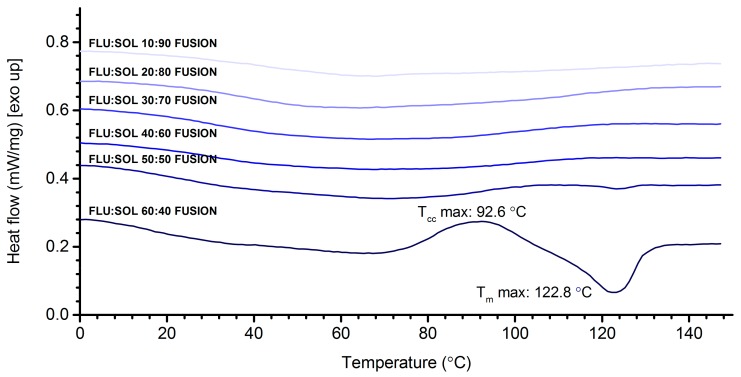
DSC curves of FLU:SOL solid dispersions prepared by the fusion method after 14 days of stability studies (40 °C, 40% RH) (first heating).

DSC curves (Figure 2) of FLU:SOL polymer blends with a drug content from 10 to 40 wt. % did not display any peaks associated with melting or recrystallisation of FLU after accelerated stability studies. This indicated increased thermal stability of the amorphous drug incorporated in the polymer matrix. The composite containing 50 wt. % of the drug displayed a low intensity broad endothermic peak starting at 113 °C, which could be assigned to the melting of the crystalline FLU. The DSC curve of 60:40 FLU:SOL blend displayed an intense exothermic peak centred at 92.6 °C followed by broad melting endotherm with a maximum at 122.8 °C similar to the starting material (Figure S7). These results indicated that a maximum of 40 wt. % of FLU could be incorporated in the SOL matrix to form a thermally stable amorphous solid dispersion.

#### The Effect of Elevated Humidity on the Formation of FLU Form II Embedded in the SOL Polymer Matrix and the Stability of an Amorphous Drug in Solid Dispersions

**Figure 3:**
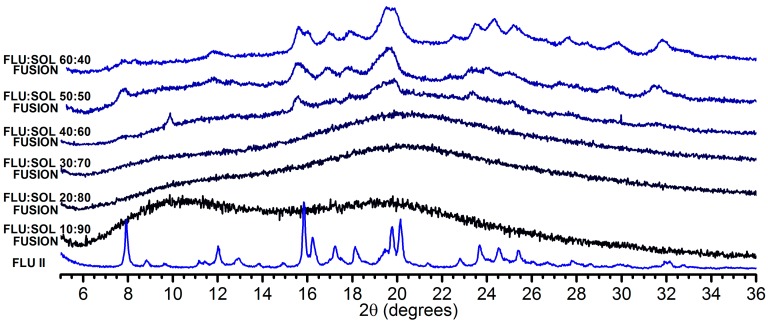
PXRD patterns of FLU:SOL solid dispersions prepared by the fusion method after 14 days of stability studies (40 °C, 70% RH).

Water can decrease physicochemical stability of amorphous solid dispersions as it can trigger the crystallisation of embedded drug molecules and induce phase separation of an API from a polymer matrix. This is due to the increased molecular mobility of an API or a polymer in amorphous systems as water can act as the plasticising agent resulting in the strong decrease of a *T*_g_ [37] or it can compete with drug molecules in hydrogen bonding with a polymer affecting noncovalent interactions responsible for increased stability of amorphous solid dispersions [38,39]. We found that FLU:SOL blends stored at 70% RH were more flexible during handling, as compared to rigid materials stored at 40% RH. This was due to the increased water content in the materials as determined using TGA analysis (Figure S13). The PXRD diffractograms of the materials containing 10–30 wt. % of FLU displayed only a diffuse ‘halo’ after 14 days of accelerated stability studies at 40 °C/70% RH. This indicated increased stability of amorphous FLU incorporated in the polymer matrix as compared to bulk drug. When FLU content within the formulations was above 40 wt. %, crystallisation of the drug to a metastable form II was observed (Figure 3). It should be noted that forms I and II of FLU were reported to crystallise to monohydrate above 40% RH [40]. We also observed spontaneous formation of FLU hydrate upon storage of FLU form I in our laboratory (ca. 22 ± 2 °C and 45–55% RH). Interestingly, we did not observe formation of FLU monohydrate in the investigated materials, which may indicate that SOL promotes formation of FLU form II regardless of the relative humidity conditions [41].

**Figure 4:**
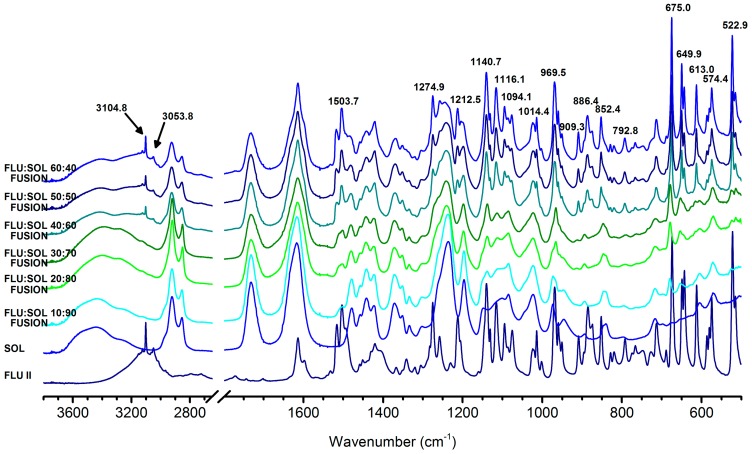
FTIR spectra of FLU:SOL solid dispersions prepared by the fusion method after 14 days of stability studies (40 °C, 70% RH).

In agreement with the PXRD results, the IR spectra (Figure 4) of the solid dispersions containing more than 30 wt. % of FLU, displayed characteristic bands of form II at: 3104.8 cm^−1^, 3053.8 cm^−1^, 1503.7 cm^−1^, 1274.9 cm^−1^, 1140.7 cm^−1^, 1016.1 cm^−1^, 969.5 cm^−1^, 909.3 cm^−1^, 886.4 cm^−1^, 852.4 cm^−1^, 675.0 cm^−1^, 649.9 cm^−1^, 613.0 cm^−1^, 522.9 cm^−1^ (vibrational band assignments Table S1).

DSC curves (Figure S14) supported by TGA results displayed broad endothermic events related to the evaporation of water from the solid dispersions. Endotherms recorded above 110 °C for the materials with a drug content above 40 wt. % could be assigned to the melting of crystalline FLU and/or dissolution of recrystallized drug in a polymer matrix.

### 3.3. Stability and Crystallisation Behaviour of Amorphous FLU in Spray Dried Solid Dispersions

#### 3.3.1. The Effect of Materials Composition on Morphology of Spray Dried Solid Dispersions

**Figure 5:**
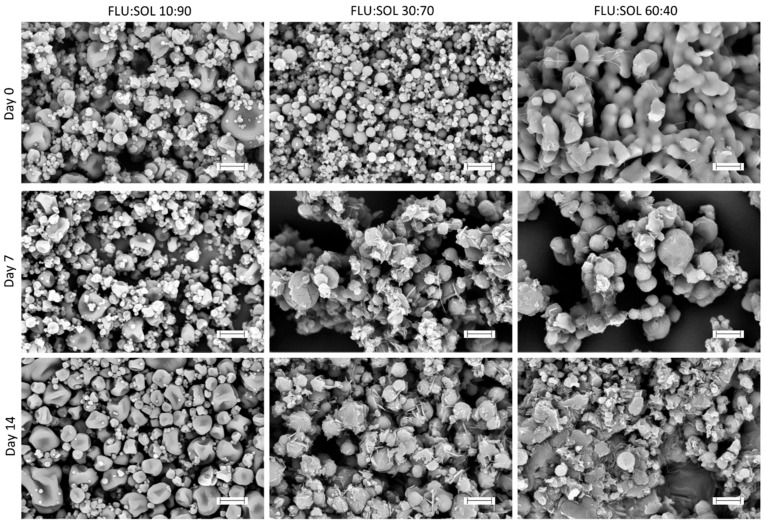
SEM microphotographs of solid dispersion obtained using a spray drying technique at Day 0 and after an accelerated stability study (40 °C, 40% RH, scale bar 10 µm).

The morphology of spray dried materials was evaluated using SEM directly after the preparation (Day 0) and after 7 and 14 days of storage at 40 °C/40% RH. The differing composition of the materials (i.e., FLU:SOL ratio) resulted in different morphologies of obtained particles (Figure 5). The FLU:SOL 10:90 formulation was composed of the two types of particles i.e., small (ca. 1–2 μm in diameter) round particles with smooth surfaces and large (ca. 5–15 μm in diameter) round particles with concave surfaces. The material composed of 30 wt. % FLU displayed uniform round-shaped particles (ca. 1–5 μm in diameter) with smooth surfaces, while the 60:40 FLU:SOL formulation comprised of large interconnected particles with smooth surfaces. Furthermore, the SEM images of the material containing 60 wt. % of FLU displayed small needles of the crystalline FLU in agreement with PXRD and FTIR analysis (see Section 3.3.2). Upon storage, the materials containing 10 wt. % of FLU did not show any signs of drug crystallisation after 7 and 14 days at 40 °C/40% RH, while the SEM images of the FLU:SOL 30:70 and 60:40 formulations displayed growth of the FLU crystals. These were observed as needles on the surfaces and plate-like crystals growing from the centre of the particles.

#### 3.3.2. The Effect of the Drug:Polymer Ratio on Stability and Crystallisation Behaviour of an Amorphous FLU in Spray Dried Solid Dispersions

**Figure 6:**
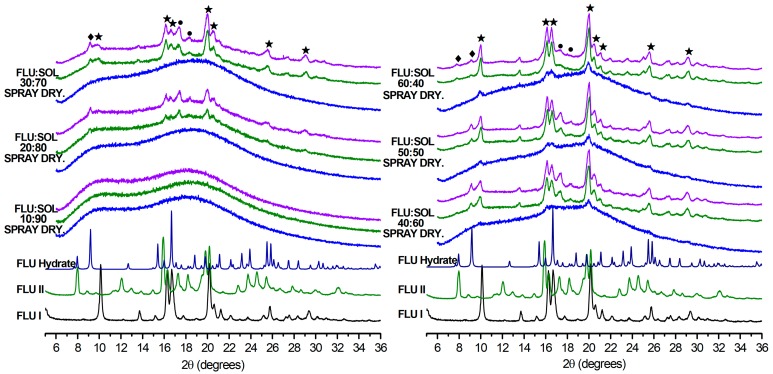
PXRD patterns of FLU:SOL solid dispersion obtained using spray drying directly after the preparation (blue) and after 7 (green) and 14 (purple) days of storage at 40 °C, 40% RH (★ FLU form I, ● FLU form II, ♦ FLU hydrate).

The spray dried FLU:SOL materials with a drug content below 30 wt. % investigated immediately after preparation did not display peaks of the crystalline FLU in the PXRD patterns, while the peaks of FLU form I were detected in the materials with a drug content above 40 wt. % (Figure 6). After the first week of accelerated stability studies only the 10:90 FLU:SOL formulation stayed amorphous. Concomitant crystallisation of polymorphs I, II and the hydrate were observed in the solid dispersions containing more than 20 wt. % of the drug. Furthermore, based on the relative intensity of the PXRD peaks, preferential crystallisation of FLU form I in the obtained materials was detected during storage. It needs to be emphasized that based on the TGA measurements water content in the spray dried FLU:SOL materials after 14 days of accelerated ageing was below 0.5% *w*/*w* (Figure S15).

**Figure 7:**
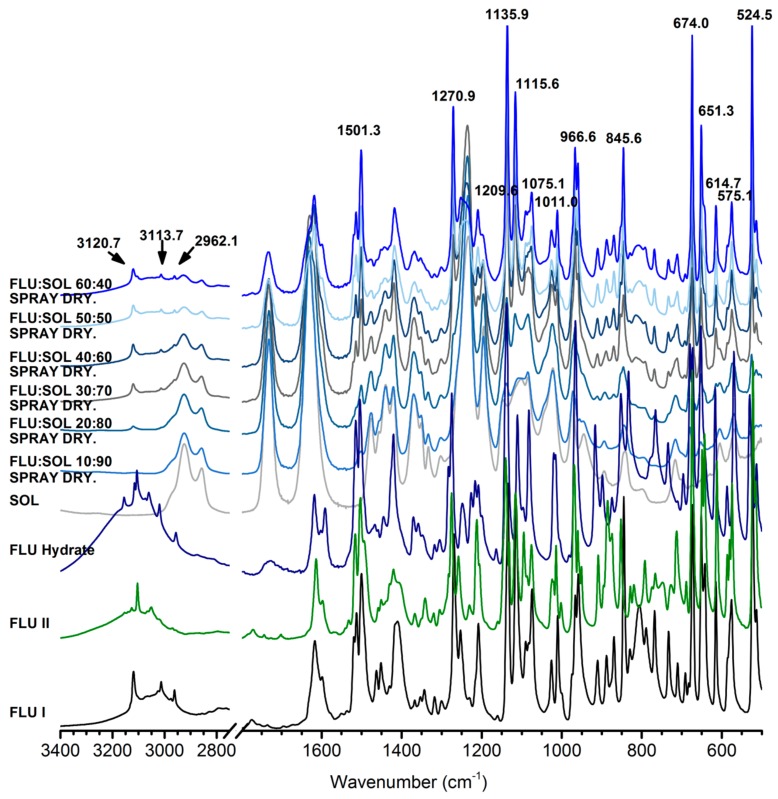
FTIR spectra of FLU:SOL solid dispersions prepared by spray drying after 14 days of stability studies (40 °C, 40% RH).

FTIR spectra recorded immediately after the preparation of spray dried solid dispersions confirmed the presence of amorphous FLU in the materials containing 10 and 20 wt. % of a drug. At 30–60 wt. % of FLU (Figure S16) characteristic peaks of forms I at 3121.2 cm^−1^ and II or hydrate 3105.8 cm^−1^ were observed. Several other peaks of the crystalline FLU observed in the spectra could not be unequivocally assigned to either of the forms (I, II or FLU hydrate) due to overlapping peaks. The FTIR results obtained after 14 days of accelerated stability studies indicated preferential formation of FLU form I in spray dried composites (Figure 7). Increasing drug content peaks at 3120.7 cm^−1^, 3113.7 cm^−1^ and 2962.1 cm^−1^ were attributed to the form I proportionally emerging from the baseline. However at lower wavenumbers, peaks at 1501.3 cm^−1^, 1270.9 cm^−1^, 1209.6 cm^−1^, 1135.9 cm^−1^, 1115.6 cm^−1^, 1075 cm^−1^, 1011.0 cm^−1^, 966.6 cm^−1^, 845.6 cm^−1^, 674.0 cm^−1^, 651.3 cm^−1^, 614.7 cm^−1^, 575.1 cm^−1^, 524.5 cm^−1^ diverged ca. 1–2 cm^−1^ from untreated form I what could be explained by the presence of polymorph II and residues of an amorphous FLU in the samples.

**Figure 8:**
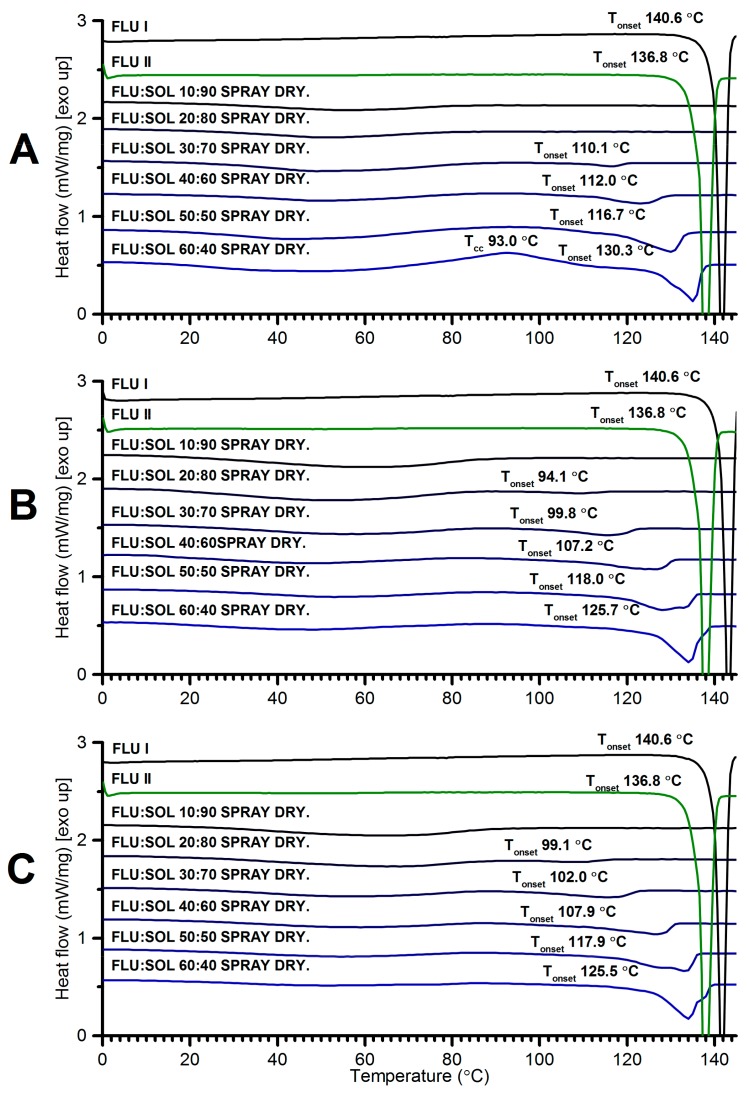
DSC curves of FLU:SOL solid dispersions prepared by spray drying and analysed directly after preparation (**A**) and after 7 (**B**) and 14 (**C**) days of storage at 40 °C/40% RH.

With the increasing content of SOL in the materials broadening and a decrease in the melting point of FLU was detected in the DSC curves (Figure 8). This may be related to partial dissolution of a drug in a polymer matrix prior to melting and/or formation of nanosize crystals [34]. The lack of endothermic events above 100 °C in the FLU:SOL 10:90 formulation confirmed stabilization of an amorphous FLU embedded in the SOL matrix. An exothermic peak at ca. 93 °C observed for the freshly prepared material containing 60 wt. % of FLU could be assigned to cold crystallisation of the FLU form II prior to melting at 130.3 °C in agreement with crystallisation behaviour of the neat amorphous FLU. After 7 and 14 days of storage the DSC curves of the solid dispersions containing 50 and 60 wt. % of FLU displayed two melting peaks between 125–140 °C of two crystalline phases in agreement with the FTIR and PXRD results (Figure 6, Figure 7 and Figure 8).

Similar to the solid dispersions obtained using the fusion method, the DSC curves of the FLU:SOL spray dried materials recorded during the second heating cycle revealed a single *T*_g_ per system, indicating miscibility of both components and the existence of a single amorphous phase (see ESI Figure S10). The experimentally determined *T*_g_ values of the spray dried materials were in excellent agreement with the *T*_g_ values recorded for the FLU:SOL blends obtained using the fusion method (see ESI Figures S10 and S11). The experimental *T*_g_ values of the composites with a drug content above 20 wt. % displayed negative deviation from the glass transition temperatures calculated using the Gordon-Taylor equation, indicating nonideal mixing of both components regardless of the preparation method.

#### 3.3.3. The Effect of the Material Composition on the Dissolution Rate of Spray Dried Solid Dispersions

**Figure 9:**
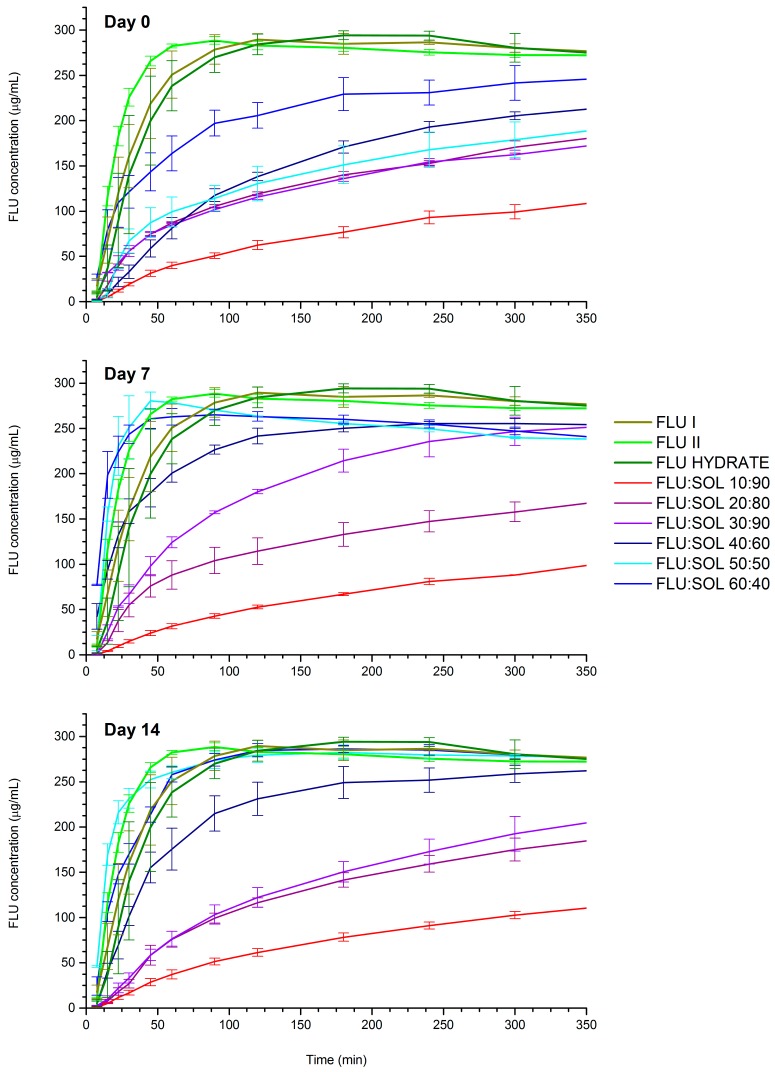
Dissolution profiles of pure FLU and spray dried FLU:SOL solid dispersions immediately after preparation and after 7 and 14 days at 40 °C/40% RH. Data are shown as mean ± SD (*n* = 3).

The dissolution studies of FLU solid forms demonstrated that FLU form II had the highest dissolution rate across evaluated polymorphs followed by FLU form I and the FLU hydrate, which showed only minor differences in the dissolution rate. The freshly prepared spray dried FLU:SOL solid dispersions displayed a slower dissolution rate as compared to the crystalline FLU forms I and II (Figure 9). This could be attributed to the slow diffusion of drug molecules through a thick polymer gel formed on the particles and devitrification of amorphous drug and recrystallisation to FLU hydrate in contact with an aqueous environment [42]. Similar observations have been reported for capecitabine, felodipine, celecoxib and diazepam [43,44,45]. The dissolution rate of the analysed materials changed significantly after storage at 40 °C/40% RH. The formulations containing 50–60 wt. % of FLU after 7 and 14 days of accelerated stability studies displayed an increased dissolution rate as compared to the starting materials. This can be related to increased crystallinity of the analysed materials as determined using PXRD and FTIR analysis and formation of FLU form II within the polymer matrix, which displayed the highest dissolution rate across the evaluated FLU forms (I, II and hydrate). On the contrary, the dissolution rate of FLU:SOL 10:90 and 20:80 formulations did not change upon storage, while the material containing 30 wt. % of FLU displayed only minor change in the dissolution rate after 7 days of storage at 40 °C/40% RH. These results indicate that the drug:carrier ratio is one of the main factors controlling the dissolution rate of FLU embedded in the SOL polymer matrix.

## 4. Conclusions

The addition of polymers as excipients in pharmaceutical solid dispersions can stabilise the amorphous state of a drug or direct its crystallisation towards new or metastable polymorphs. The crystallisation outcome or extended stabilisation of ASDs depends on the polymer structure, properties, its content in the formulation as well as the preparation technique. In this study, we synthesised solid dispersions of FLU and SOL using fusion and spray drying techniques and investigated the crystallisation pathways of amorphous FLU embedded in the polymer matrix during storage at 40 °C and 40/70% RH. Preferential crystallisations towards metastable FLU form II were observed at 40 °C and 70% RH for the materials obtained using the fusion method with FLU content above 40 wt. %. The FLU:SOL dispersions with a drug content below 30 wt. % stayed amorphous during 14 days of accelerated stability studies (40 °C/70% RH) as confirmed using PXRD and FTIR. Interestingly, we did not observe the formation of FLU monohydrate in the investigated materials obtained using the fusion method, which may indicate that Soluplus promotes formation of FLU form II regardless of the relative humidity conditions. In contrast to the fully amorphous materials obtained using the fusion method, the crystalline FLU was detected in the spray dried materials investigated directly after preparation at a drug content above 30 wt. %. Furthermore, the formation of a mixture of FLU forms I, II and hydrate was observed for the materials stored at 40 °C/40% RH for 7 and 14 days, with preferential crystallisation towards form I over time. Only the FLU:SOL 10:90 formulation stayed amorphous after 14 days of an accelerated stability study. This study presents the effect of polymer addition and different formulation techniques on the crystallisation pathways and stability of model drug (FLU) embedded in the amphiphilic polymer matrix composed of Soluplus. The presented results are of importance for the controlled crystallisation of metastable polymorphs and knowledge-based design of successful amorphous solid dispersions.

## Supplementary Materials

The following are available online at https://www.mdpi.com/1999-4923/12/1/12/s1, data from stability studies of amorphous FLU; PXRD, FTIR, DSC and TGA data of FLU:SOL dispersions obtained using the fusion method directly after preparation and after 14 days at 40 °C/40% RH; PXRD data of FLU:SOL 60:40 crystallisation at different temperatures; assignment of the FTIR peaks for different forms of FLU. Figure S1. DSC curves of raw FLU (blue, second heating) and FLU crystallised from supercooled amorphous glass after 14 days of accelerated ageing at 40 °C, 40% RH (purple) and at 40 °C, 70% RH (black), Figure S2. PXRD patterns of FLU form I (blue), FLU hydrate (green) and FLU recrystallized from supercooled amorphous glass after 14 days of accelerated ageing at 40 °C, 40% RH (purple) and at 40 °C, 70% RH (black), Figure S3. FTIR spectra of FLU form I (blue), FLU hydrate (green) and FLU samples recrystallized from supercooled amorphous glass after 14 days of accelerated ageing at 40 °C, 40% RH (purple) and at 40 °C, 70% RH (black), Figure S4. PXRD patterns of FLU:SOL solid dispersions obtained using the fusion method recorded immediately after preparation, Figure S5. FTIR spectra of FLU:SOL solid dispersions obtained using the fusion method recorded immediately after preparation, Figure S6. FTIR spectra of raw Soluplus, Soluplus heated to 145 °C and cooled to RT (SOL fusion) and 60:40 FLU:SOL solid dispersion obtained using the fusion method, Figure S7. DSC curves of FLU:SOL solid dispersions obtained using the fusion method recorded immediately after preparation, Figure S8. TGA curves of FLU:SOL solid dispersions obtained using the fusion method recorded immediately after preparation, Figure S9. PXRD patterns of FLU:SOL 60:40 solid dispersion prepared using the fusion method after heating at 80, 100, 125 and 130 ± 2 °C for 15 min. The PXRD pattern of FLU form II was provided for comparison, Figure S10. DSC curves (zoomed T_g_ temperature region in the second heating cycle) of FLU, SOL and FLU:SOL solid dispersions prepared using the fusion (A) and spray drying (B) methods with determined T_g_ of the obtained materials, Figure S11. A. Theoretical T_g_ values calculated using the Gordon–Taylor equation and experimental T_g_ values of neat FLU, SOL and FLU:SOL solid dispersions obtained using the fusion and spray drying methods. B. The difference between experimentally obtained T_g_ values of FLU:SOL solid dispersions and theoretically calculated T_g_ of FLU:SOL binary mixtures using the Gordon–Taylor equation, Figure S12. PXRD patterns of FLU:SOL solid dispersions prepared using the fusion method after 14 days of stability studies (40 °C, 40% RH), Figure S13. TGA curves of FLU:SOL solid dispersions obtained using the fusion method recorded after 14 days of stability studies (40 °C, 70% RH), Figure S14. DSC curves of FLU:SOL solid dispersions prepared using the fusion method after 14 days of stability studies (40 °C, 70% RH), Figure S15. TGA curves of FLU:SOL solid dispersions obtained using the spray drying method recorded after 14 days of stability studies (40 °C, 40% RH), Figure S16. FTIR spectra of FLU:SOL solid dispersions obtained using the spray drying method acquired immediately after preparation, Table S1. FTIR vibrational bands assignments of FLU forms I, II, amorphous FLU, FLU hydrate and melted FLU after accelerated stability studies at 40 °C and 40/70% RH.
