# Supplementary material for: Stabilisation and Growth of Metastable Form II of Fluconazole in Amorphous Solid Dispersions
Source: Pharmaceutics. 2019 Dec 20;12(1):12. doi: 10.3390/pharmaceutics12010012 (PMC7023302; doi:10.3390/pharmaceutics12010012)
Supplement: Supplementary file 1 [file pharmaceutics-12-00012-s001.pdf]

pharmaceutics

Supplementary Materials: Stabilisation and Growth of Metastable Form II of Fluconazole in Amorphous Solid Dispersions

Maciej Nowak, Maciej Gajda, Przemysław Baranowski, Patrycja Szymczyk, Bożena Karolewicz and Karol P. Nartowski

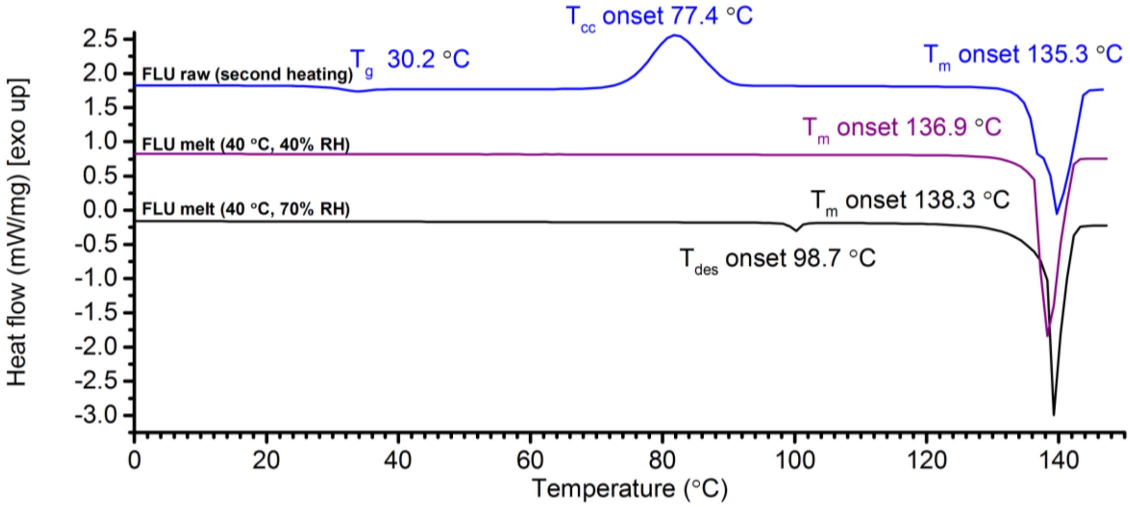

Figure S1. DSC curves of raw FLU (blue, second heating) and FLU crystallised from supercooled amorphous glass after 14 days of accelerated ageing at 40 °C, 40% RH (purple) and at 40 °C, 70% RH (black).

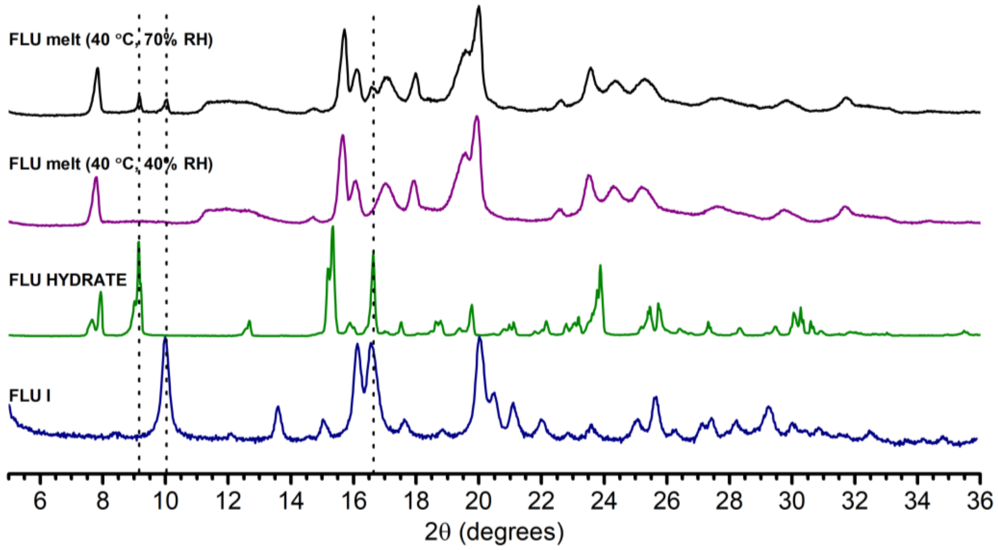

Figure S2. PXRD patterns of FLU form I (blue), FLU hydrate (green) and FLU recrystallized from supercooled amorphous glass after 14 days of accelerated ageing at 40 °C, 40% RH (purple) and at 40 °C, 70% RH (black).

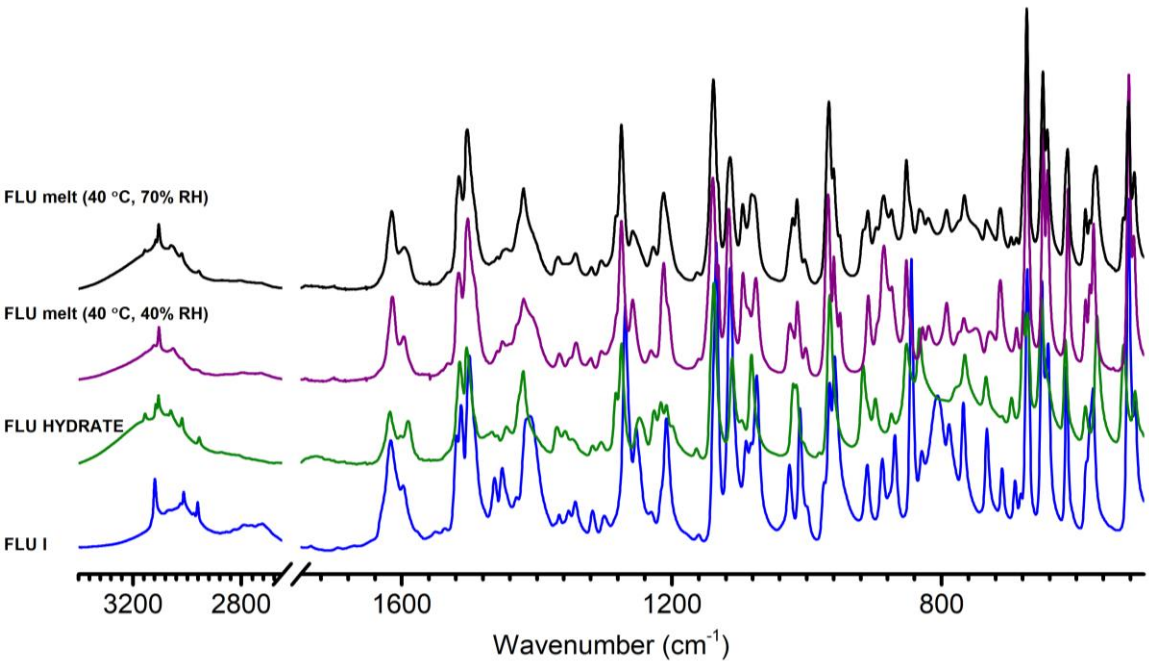

Figure S3. FTIR spectra of FLU form I (blue), FLU hydrate (green) and FLU samples recrystallized from supercooled amorphous glass after 14 days of accelerated ageing at 40 °C, 40% RH (purple) and at 40 °C, 70% RH (black).

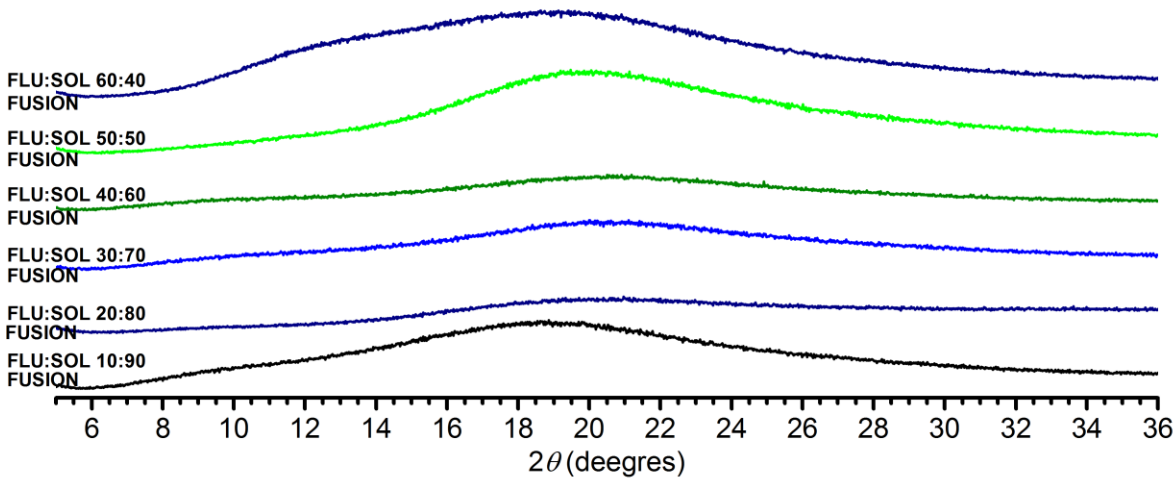

Figure S4. PXRD patterns of FLU:SOL solid dispersions obtained using fusion method recorded immediately after preparation.

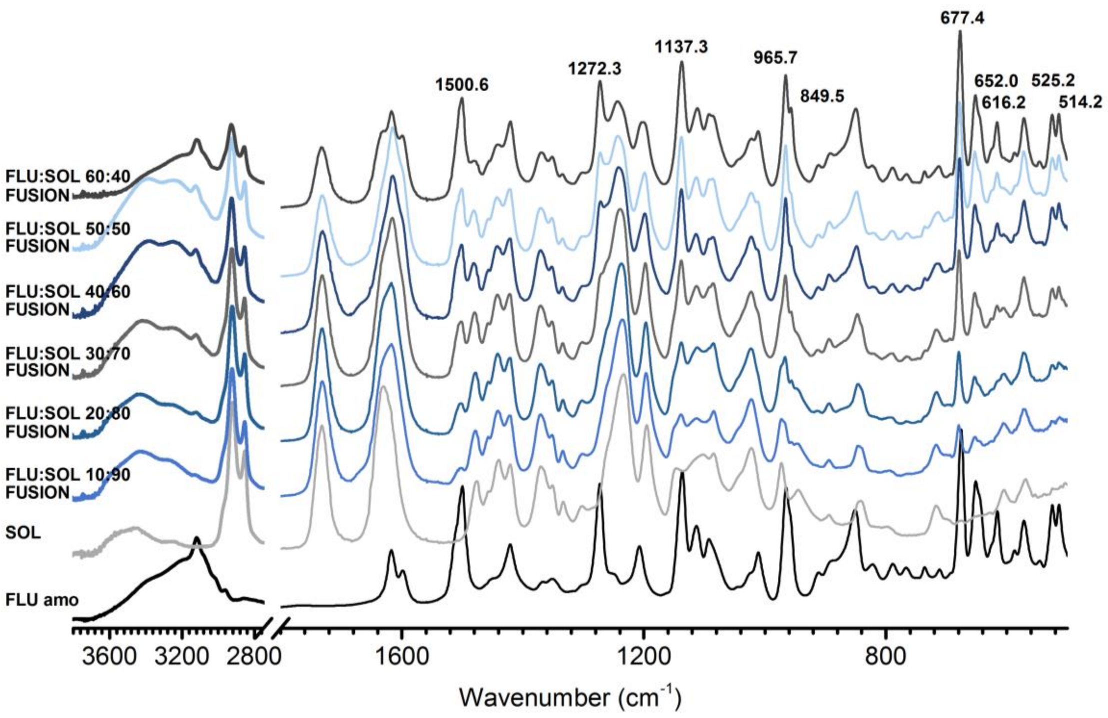

Figure S5. FTIR spectra of FLU:SOL solid dispersions obtained using fusion method recorded immediately after preparation.

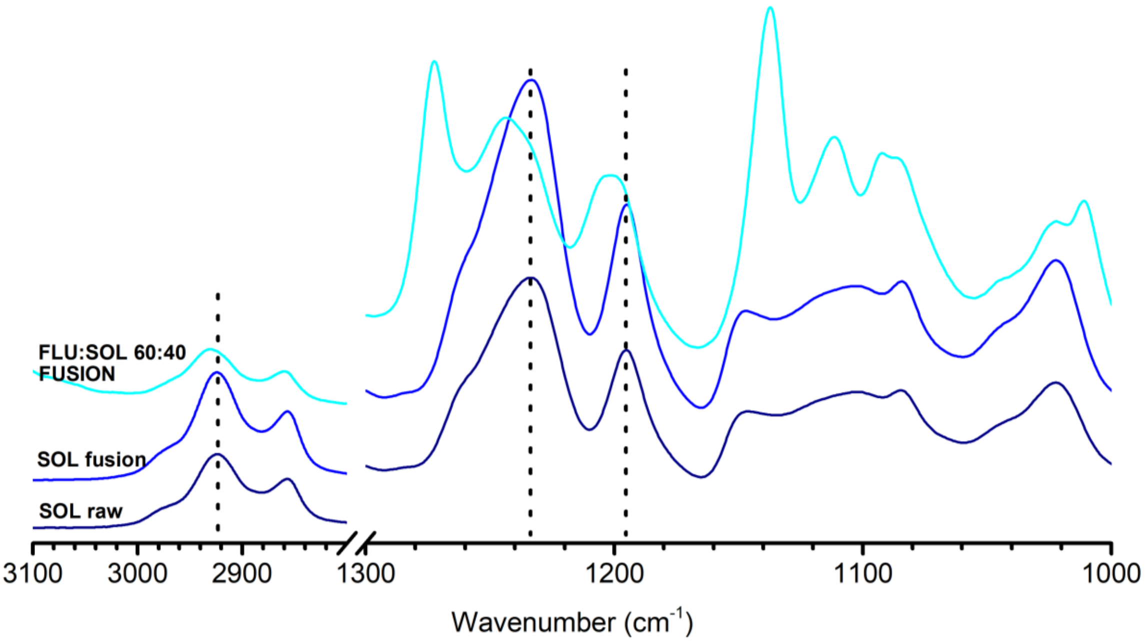

Figure S6. FTIR spectra of raw Soluplus, Soluplus heated to 145 °C and cooled to RT (SOL fusion) and 60:40 FLU:SOL solid dispersion obtained using fusion method.

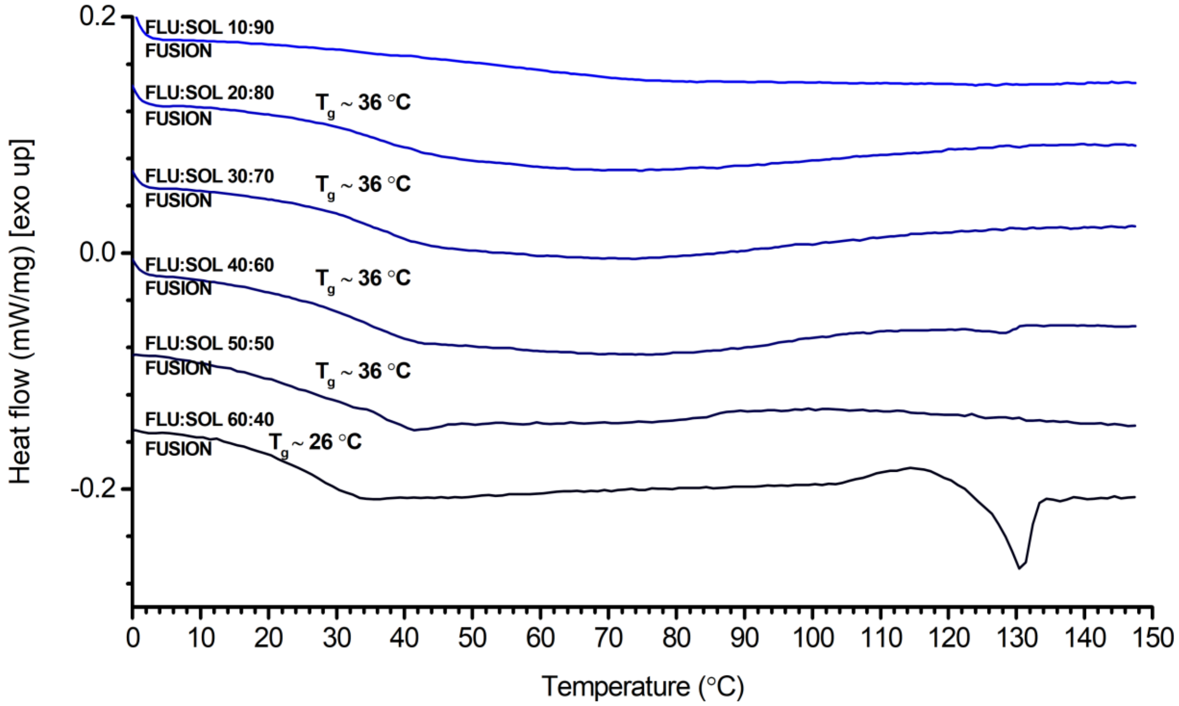

Figure S7. DSC curves of FLU:SOL solid dispersions obtained using fusion method recorded immediately after preparation.

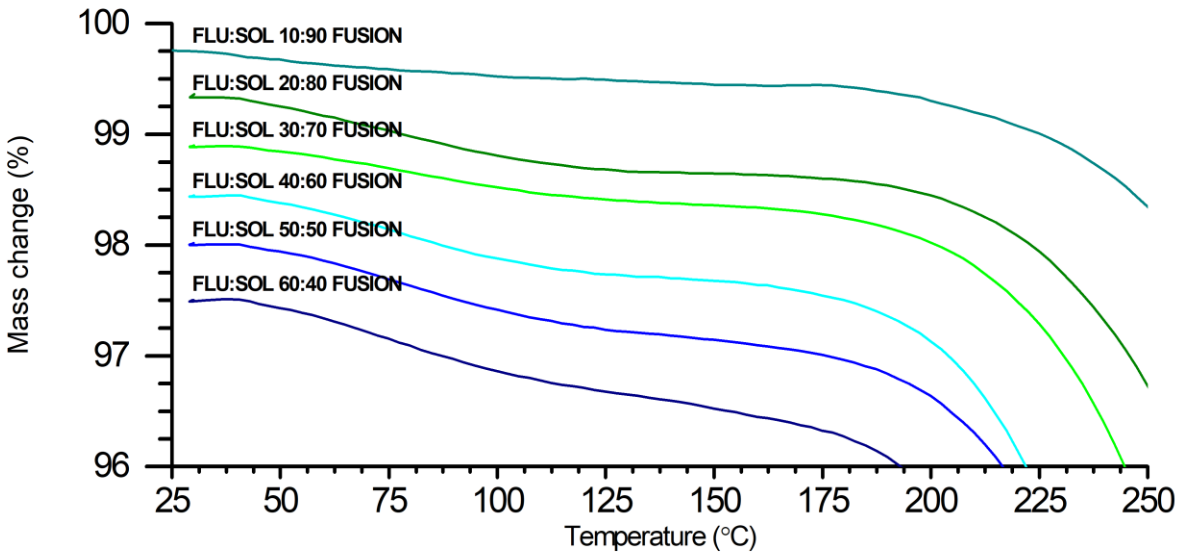

Figure S8. TGA curves of FLU:SOL solid dispersions obtained using fusion method recorded immediately after preparation.

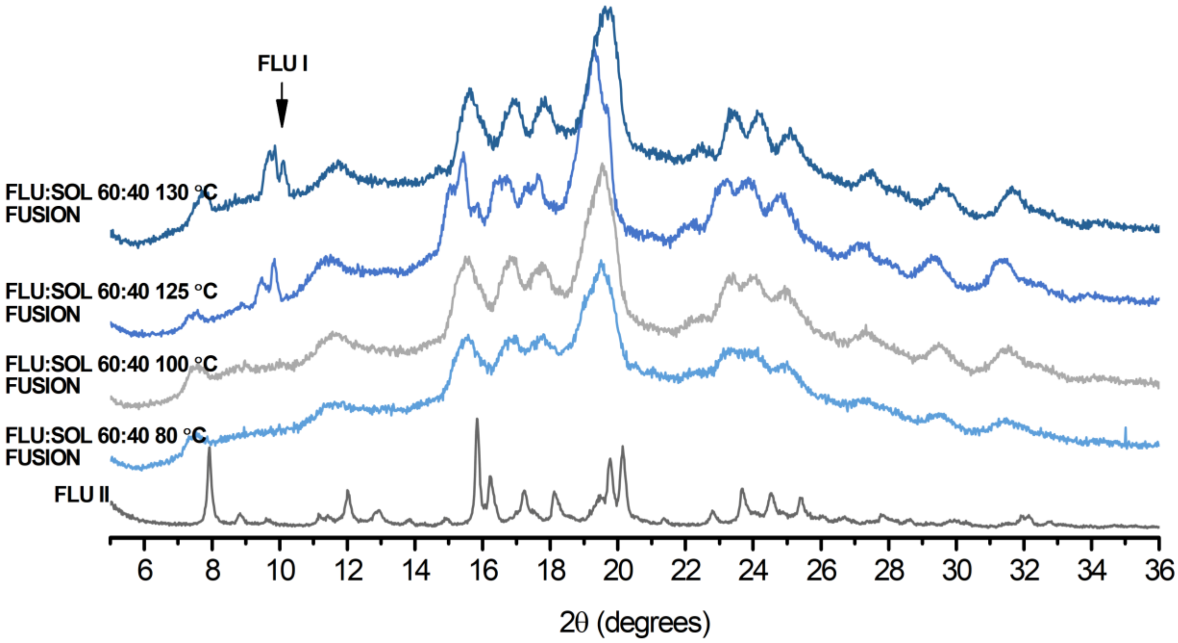

Figure S9. PXRD patterns of FLU:SOL 60:40 solid dispersion prepared using fusion method after heating at 80, 100, 125 and 130 ± 2 °C for 15 minutes. The PXRD pattern of FLU form II is provided for comparison.

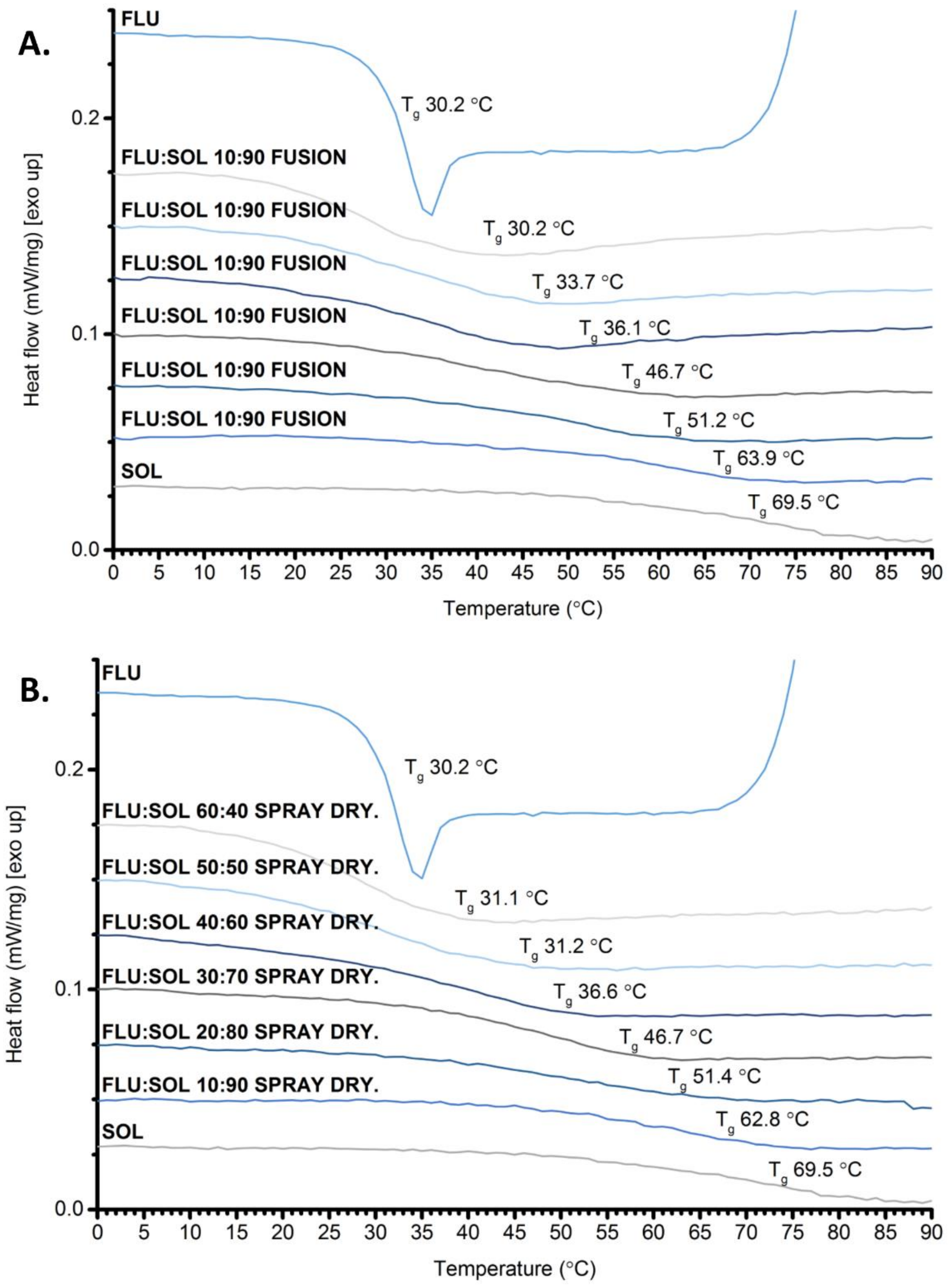

Figure S10. DSC curves (zoomed T_g temperature region in the second heating cycle) of FLU, SOL and FLU:SOL solid dispersions prepared using fusion (**A**) and spray drying (**B**) method with determined T_g of the obtained materials.

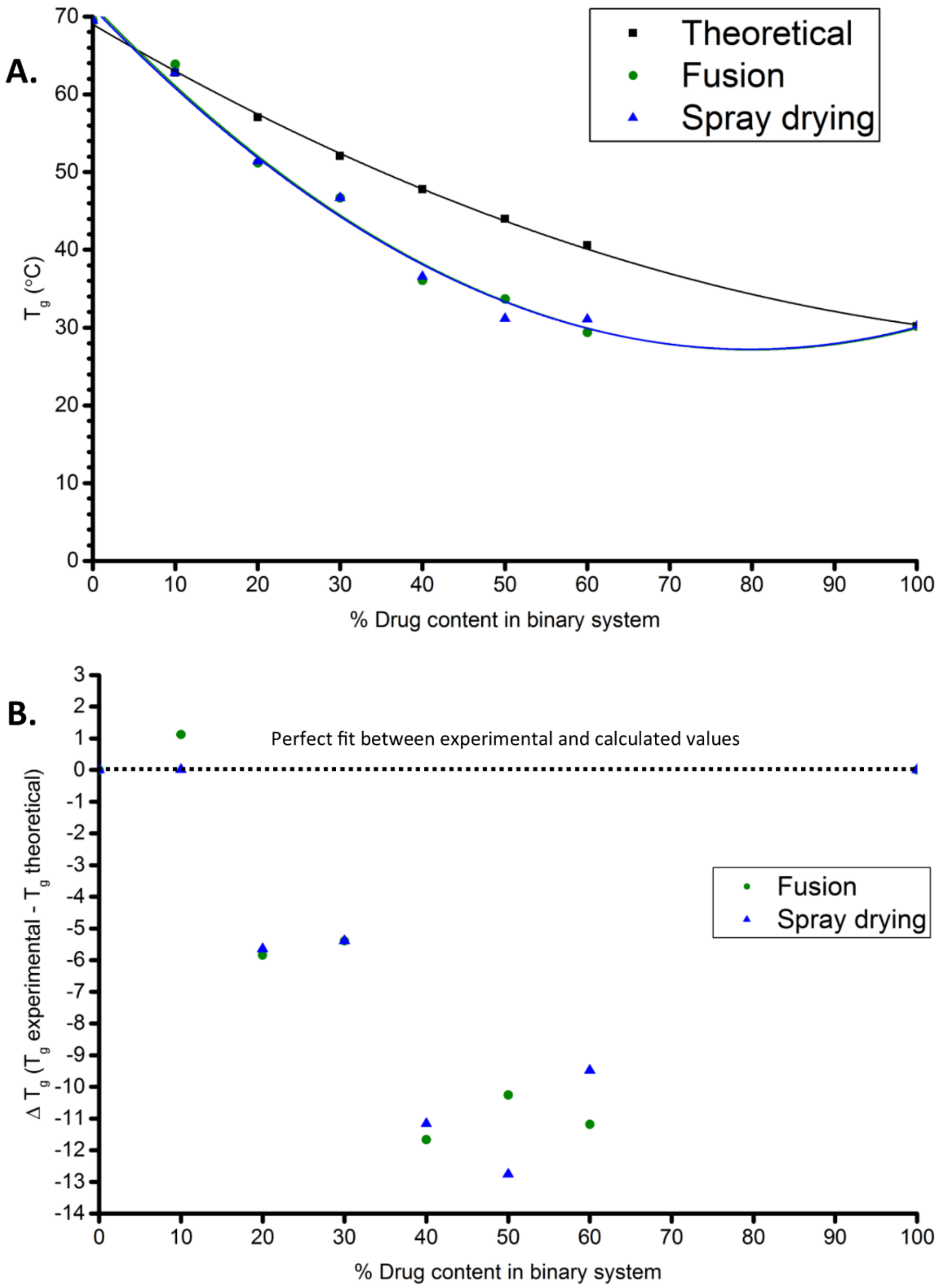

Figure S11. (**A**) Theoretical T_g values calculated using Gordon-Taylor equation and experimental T_g values of neat FLU, SOL and FLU:SOL solid dispersions obtained using fusion and spray drying methods. (**B**) The difference between experimentally obtained T_g values of FLU:SOL solid dispersions and theoretically calculated T_g of FLU:SOL binary mixtures using Gordon-Taylor equation.

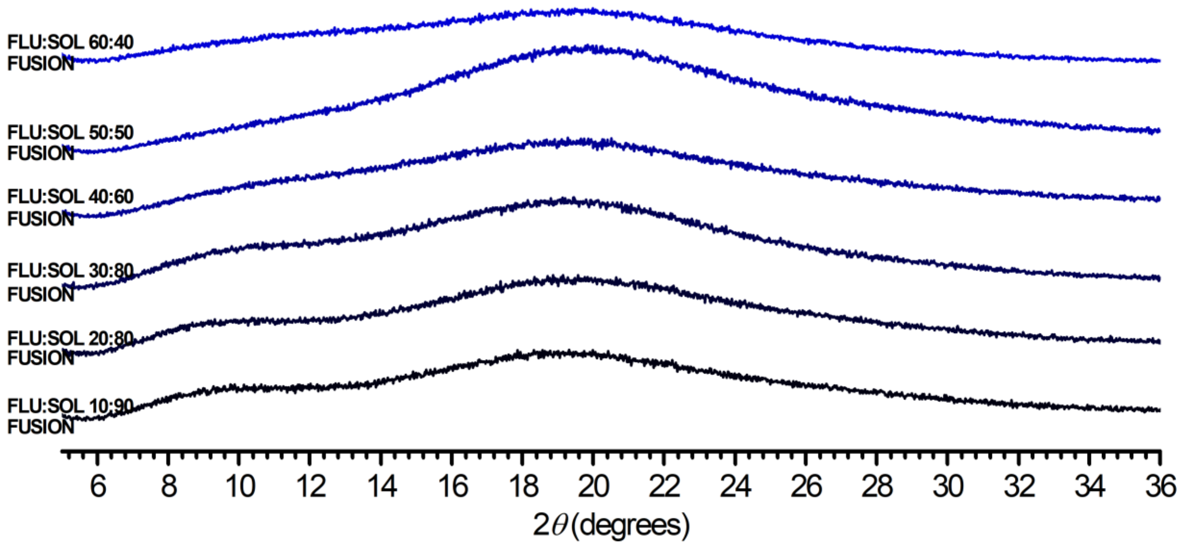

Figure S12. PXRD patterns of FLU:SOL solid dispersions prepared using fusion method after 14 days of stability studies (40 °C, 40% RH).

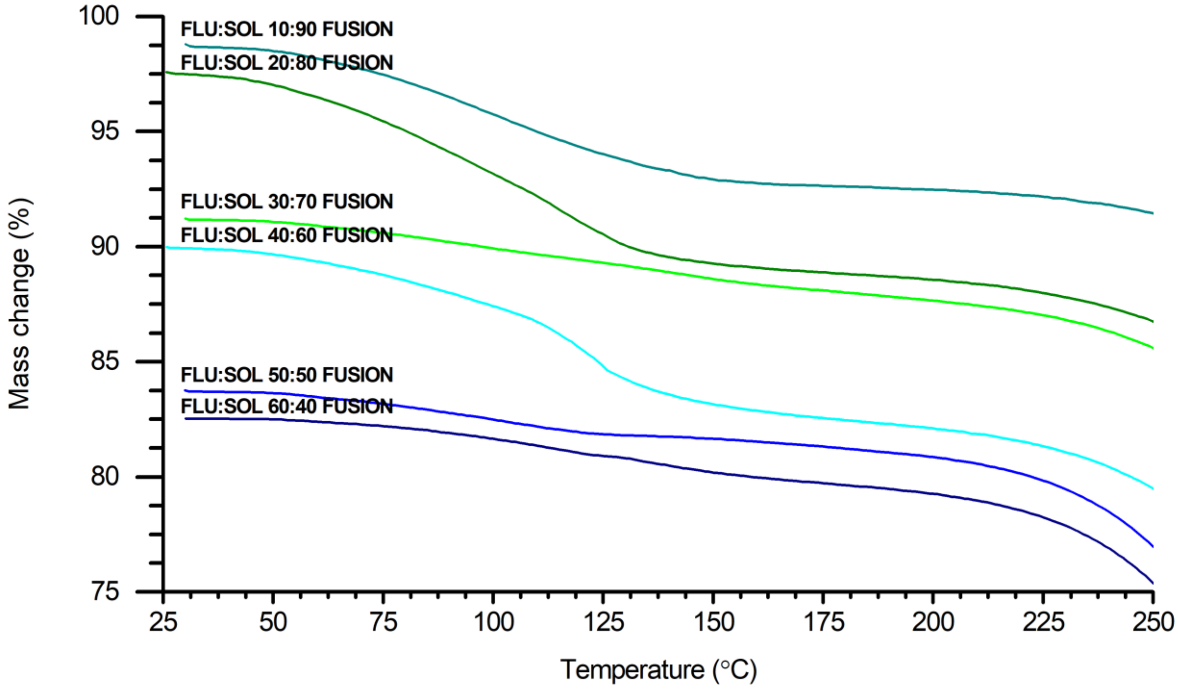

Figure S13. TGA curves of FLU:SOL solid dispersions obtained using fusion method recorded after 14 days of stability studies (40 °C, 70% RH).

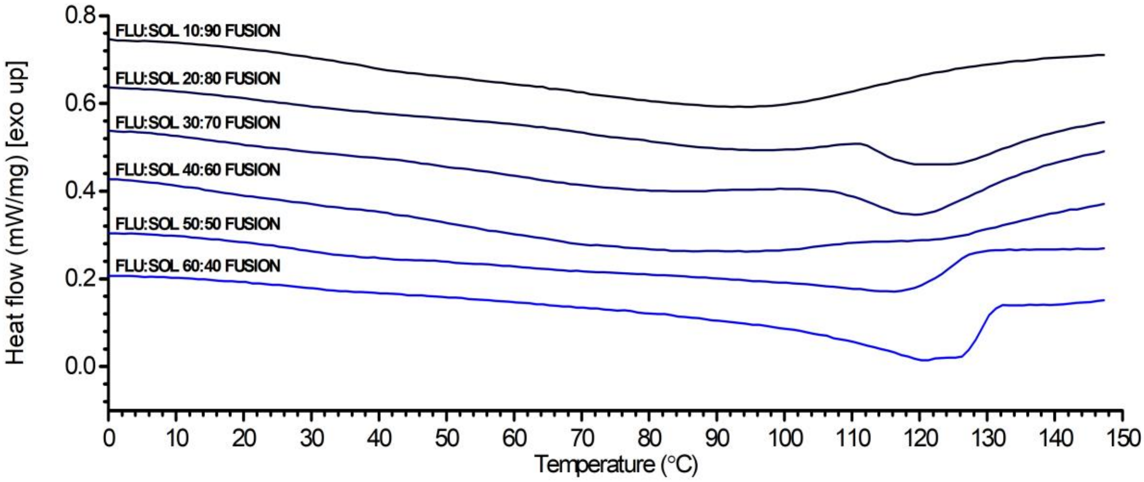

Figure S14. DSC curves of FLU:SOL solid dispersions prepared using fusion method after 14 days of stability studies (40 °C, 70% RH).

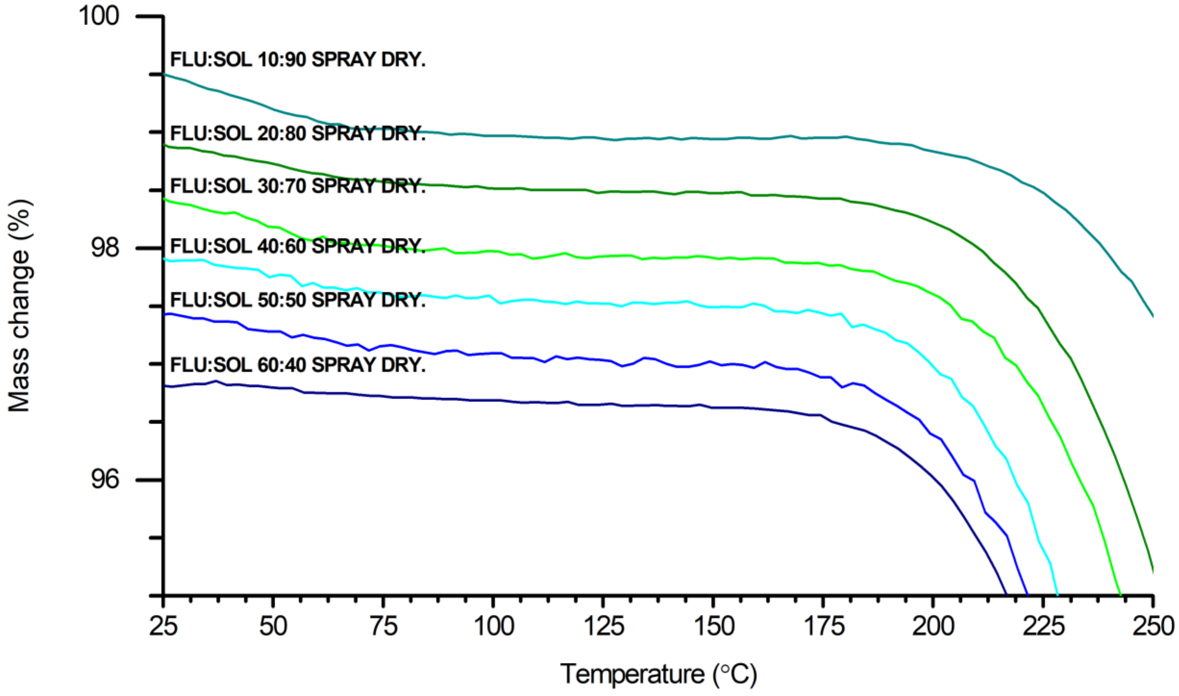

Figure S15. TGA curves of FLU:SOL solid dispersions obtained using spray drying method recorded after 14 days of stability studies (40 °C, 40% RH).

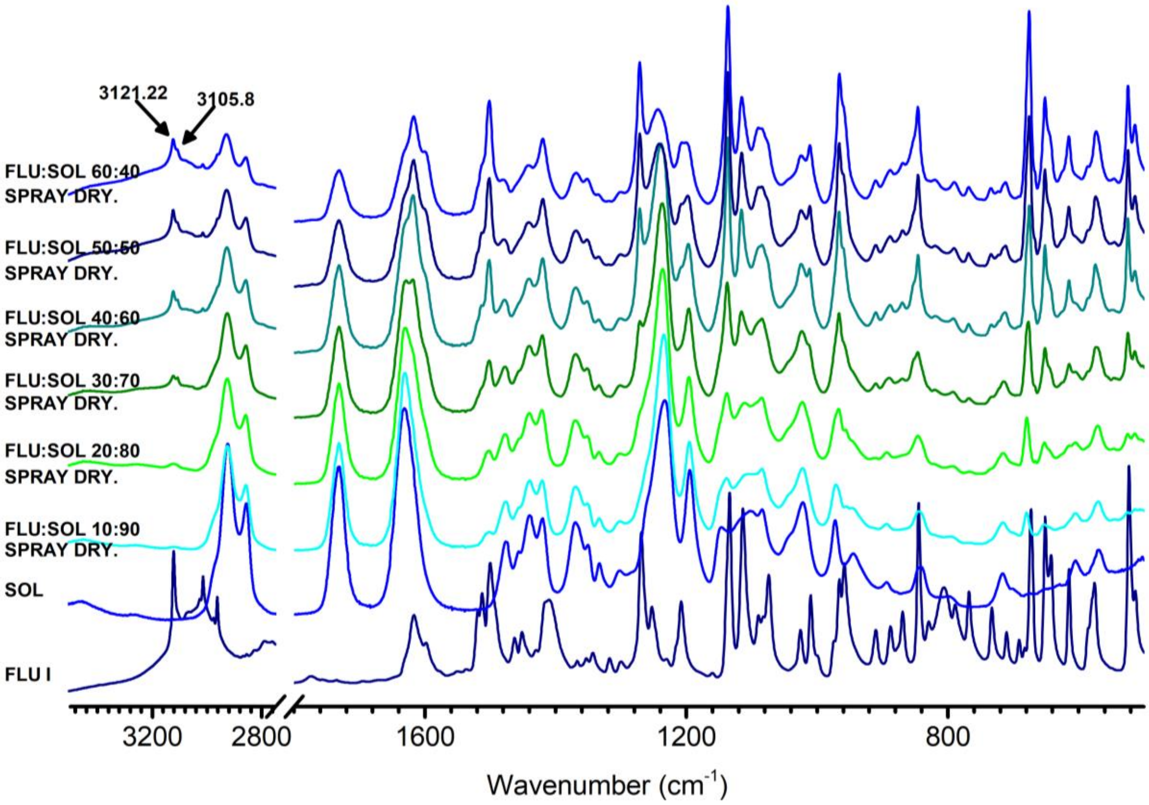

Figure S16. FTIR spectra of FLU:SOL solid dispersions obtained using spray drying method acquired immediately after preparing.

Table S1. FTIR vibrational bands assignments of FLU forms I, II, amorphous FLU, FLU hydrate and melted FLU after accelerated stability studies at 40 °C and 40 / 70% RH.

Vibrational Band Assignments	FLU Form I	FLU Form II	FLU Amorphous	FLU Hydrate	Melted FLU Stored at 40 °C and 40% RH	Melted FLU Stored at 40 °C and 40% RH
Triazole C-H stretching	3019.8	3126.0, 3104.3		3115.4, 3106.3	3122.7, 3104.6	3016.1, 3105.5
Difluorophenyl CH stretching, (involved C-H···O bonding)	3013.2	3051.8		3060.9, 3020.0	3052.76	3060.2, 3020.9
Methylene C-H asymmetric stretching	2962.12			2956.8		2956.3
Difluorophenyl ring C=C stretching	1617.0	1614.5	1617.0	1618.0	1614.4	1615.5
Difluorophenyl C=C stretching	1598.7	1598.2	1598.22	1591.0	1597.7	1597.0
Triazole C=N stretching	1519.6, 1512.8, 1500.3	1516.7, 1503.7	1499.6	1514.8, 1504.7	1516.7, 1502.7	1516.0, 1503.5
Difluorophenyl ring C-C stretching	1463.22			1467.1		
Methylene scissoring	1451.6	1450.7		1445.4	1451.4	1445.6
Methylene scissoring or triazole C-N stretching	1410.2	1420.3	1420.8	1420.3	1419.8	1419.8
Methylene wagging	1367.3, 1353.3	1367.3	1366.8, 1351.4	1370.6, 1358.6	1367.0	1368.7

**Triazole C-N stretching**	1343.2, 1317.6, 1300.2	1341.7, 1321.0, 1304.6		1348.0, 1317.6, 1305.1	1342.2, 1320.0, 1304.6	1343.2, 1319.5, 1304.8
**O-H in-plane-bending (C-OH scissoring)**	1269.4	1275.2	1272.8	1274.7	1274.9	1275.0
**C-F stretching**	1253.0	1258.3		1248.2	1258.3	1258.3
**C-C stretching, difluorophenyl in plane banding (H-C=C)**	1230.8	1230.8		1216.4	1231.0	1227.7
**Triazole N-N stretching, Methylene twisting**	1208.2	1212.5	1207.2	1208.18	1212.0	1212.3
**Difluorophenyl ring C-H-in-plane bending**	1133.9, 1113.7	1140.2, 1131.5, 1115.6	1136.3, 1113.2	1137.8, 1110.8	1139.5, 1131.3	1138.5, 1113.9
**C-O-H bending, C-C-C trigonal bending**	1025.9	1022.0		1020.1	1025.0	1020.1
**Triazole N=C-N bending**	1010.0	1014.9	1011.0	1015.3	1114.1	1014.6
**Triazole N-N stretching, methylene rocking**	968.0	966.2	965.2	965.7	968,5	967.3
**C-C-C ring breathing**	959.9	958.9			959,9	960.4
**Triazole C-N=C scissoring, difluorophenyl out-of-plane-bending**	910.2	909.3	911.2	916.5	908,8	909.5
**C-O bending, triazole in-plane-bending, methylene rocking**	888.0	885.7	887.6	898.18	885.6	885.9
**Triazole ring out-of-plane-bending**	869.3	875.0		874.6	874.1	874.3
**Difluorophenyl C-H out-of-plane-bending**	845.15	851.9	850.9	852.4	852.4	852.4
**Triazole ring out-of-plane-bending**	829.7	829.7, 819.6	822.0	833.1	828.5, 819.6	833.11, 820.8
**Difluorophenyl C-H out-of-plane-bending**	806.6, 789.2	792.6	788.7		792.6	792.8
**C-N asymmetric stretching (skeleton), C-C asymmetric stretching (skeleton), triazole bending N-C-C**	768.0	766.0	766.5	766.1	767.3	766.5
**C-N symmetric stretching (skeleton), triazole bending (N=C-N)**	732.8	728.5	735.7	734.3	728.5	733.8
**C-C-C in plane bending**	710.6, 691.4, 673.0	713.0, 688.9, 674.0	711.1, 675.4	679.3, 673.0	713.0, 688.9, 674.0	713.1, 697.1, 688.9, 674.0
**Triazole ring out-of-plane-bending**	651.3, 642.2, 614.7	649.4, 643.1, 612.8	651.3, 615.7	652.3, 616.6	649.4, 643.4, 612.5	649.9, 643.6, 613.5
**C-C-C in plane bending**	585.3	586.7	587.2	587.2	586.7	587.0
**C-F in-plane-bending**	575.7	573.7	571.8	569.9	574.9	571.3
**N-C-C bending**	523.1	522.1	525.0	530.3	522.6	523.1
**C-C-C out-of-plane-bending**	513.5	515.9	513.9	513.5	515.4	514.4
